# Carbonized Composites Containing Silica Aerogels with Enhanced Hydrophobicity and Thermal Insulation via Glass Fiber and Hollow Microsphere Reinforcement

**DOI:** 10.3390/gels12050439

**Published:** 2026-05-17

**Authors:** Yuquan Cao, Ruliang Li, Zikang Chen, Miao Liu, Yumin Duan, Shuai Li, Zhi Li

**Affiliations:** 1 School of Resources and Safety Engineering, Central South University, Changsha 410083, China; 8210240801@csu.edu.cn (Y.C.);; 2 Changsha Branch, Xiandai Investment Co., Ltd., Changsha 410004, China

**Keywords:** silica aerogel-containing composites, thermal stability, hollow glass microspheres, hydrophobicity, thermal insulation

## Abstract

Facing the increasingly severe energy challenges and environmental problems, the development of thermally stable, lightweight, and thermal insulating materials is critical. Herein, we report an organic-inorganic composite strategy combined with a high-temperature carbonization step to fabricate aerogel-containing composites synergistically reinforced with chopped glass fibers and hollow glass microspheres. By systematically varying the ratio of acrylic emulsion to potassium silicate solution, we investigated the effects on the forming behavior, microstructure, hydrophobicity, thermal stability, and thermal insulation performance. Increasing the acrylic emulsion fraction substantially enhanced hydrophobicity, yielding a maximum water contact angle of 129.3°. Concurrently, the apparent density decreased from 0.18 g/cm^3^ to 0.09 g/cm^3^ and the thermal conductivity dropped from 57.9 mW/(m·K) to 29.0 mW/(m·K). Mechanical testing revealed that the compressive Young’s modulus decreased with increasing acrylic content, from 3.6 MPa for the purely inorganic sample to 0.55 MPa at 70% acrylic content, reflecting a trade-off between stiffness and organic-derived porosity. Microstructural characterization revealed a hierarchical porous network in which uniformly dispersed hollow glass microspheres and the aerogel-derived silica network form an efficient thermal barrier system. Thermogravimetric analysis demonstrated excellent thermal stability, with total weight loss below 5% up to 800 °C. Infrared thermography analysis showed that, after unilateral heating at 300 °C and 400 °C for 10 min, the backside surface temperature of the composites decreased as the acrylic emulsion content increased. At 300 °C, the temperature decreased from 176.1 °C for AP-1 to 151.0 °C for AP-4, while at 400 °C, it decreased from 228.5 °C to 199.3 °C. These results indicate that the composites exhibit effective thermal insulation and maintain structural stability under high-temperature exposure. Taken together, this facile and scalable approach yields these aerogel-containing composites that combine low density, low thermal conductivity, robust structural integrity, and good environmental resistance, as evidenced by a water contact angle of 129.3°, making them promising candidates for aerospace, building, and industrial high-temperature insulation applications.

## 1. Introduction

Amid the intensifying global energy shortages and rising environmental awareness, advanced thermal insulation materials that combine ultra-low heat transfer, low mass, and long-term thermal stability are urgently needed. Conventional insulators (e.g., polystyrene foam and mineral wool) remain widely used but suffer from relatively high thermal conductivity, flammability, moisture uptake, and limited durability, which restrict their applicability in high-temperature or harsh environments [1,2,3]. Moreover, organic materials typically exhibit poor high-temperature stability and thus cannot satisfy long-term high-temperature insulation requirements [4,5]. Therefore, the development of novel high-temperature-resistant insulation materials that combine low thermal conductivity, superior thermal stability, and broad applicability is an important research direction in materials science and engineering.

In recent years, silica aerogel (SA) has attracted considerable attention as an ultralightweight material with a three-dimensional nanoporous structure, offering extremely high porosity (typically more than 90%) and very low density (0.03~0.15 g/cm^3^) [6,7,8,9]. Its nanoscale pore network effectively suppresses gaseous convection and solid-state heat conduction, yielding ultra-low thermal conductivity that substantially outperforms conventional thermal insulation materials [10,11]. However, the nanoporous skeletal architecture also imparts intrinsic brittleness and poor mechanical strength, which limits its standalone use and generally necessitates compositing with other materials to enhance mechanical performance [12,13,14,15]. For instance, Liu et al. synthesized a PDMS-based aerogel layer using pearl stone fibers (MFs), hollow glass microspheres (HGM), and SA as the primary fillers, achieving excellent mechanical properties and thermal insulation performance [16]. Hollow glass microspheres are a crucial functional filler: their closed-cell hollow structure reduces the packing density, enhances the insulation performance, and optimizes the stress distribution [17]. Incorporating hollow microspheres into the aerogel matrix further reduces the material density and introduces an internal stationary air layer, which hinders heat conduction. Together with the nanoporous network and the fiber-reinforced framework of the silica aerogel, these characteristics synergistically form a multi-scale thermal barrier system [18,19].

In such composite systems, organic binders are often employed to improve film formation and interfacial compatibility. Acrylic emulsion, a widely used kind of polymeric dispersion with excellent film-forming ability, interfacial adhesion, and weather resistance, has found broad application in building facades, transportation equipment, and high-performance coatings [20,21]. However, acrylic emulsion alone exhibits notable limitations under high-temperature exposure. As an organic polymer, it undergoes thermal degradation at elevated temperatures, which can lead to significant weight loss, structural collapse, and pulverization of the matrix, thereby compromising both the mechanical integrity and fire safety. These drawbacks highlight the need for thermally stable inorganic components that can preserve structural coherence under thermal stress. A similar trade-off between hydrophobicity and fire hazard has been systematically reviewed for hydrophobic silica aerogels, where surface-grafted organic groups are identified as the primary source of thermal instability and flammability [22]. This motivates our use of a high-temperature carbonization step to reduce the combustible organic fraction in the composite. Potassium silicate, a water-soluble inorganic silicate, offers a compelling alternative. It forms a robust, non-combustible -Si-O-Si- network via polycondensation under elevated temperature or catalytic conditions, making it an ideal matrix for high-temperature applications [23,24,25]. In summary, rational coupling of the chopped glass fibers, hollow glass microspheres, acrylic emulsions, and potassium silicate within aerogel systems substantially enhances its mechanical robustness while maintaining low thermal conductivity.

In this work, we developed an organic-inorganic composite strategy to fabricate multiscale thermal-barrier composites by integrating silica aerogel particles, chopped glass fibers, and hollow glass microspheres into a tunable acrylic-emulsion-potassium silicate matrix. Chopped glass fibers act as high-temperature-stable reinforcements that bridge cracks and improve toughness while preserving porosity. Hollow glass microspheres reduce the bulk density and introduce sealed air inclusions that further impede heat transfer. The acrylic emulsion enhances the formation of the film and interfacial compatibility between the organic and inorganic phases with the aid of a silane coupling agent and a coalescent. A subsequent high-temperature carbonization step is employed to pyrolyze the organic components derived from the acrylic emulsion, thereby eliminating combustible fractions and reducing the overall heat-release potential. Unlike conventional carbonization that primarily targets electrical conductivity or the mechanical enhancement of aerogels, the thermal treatment here serves a distinct triple purpose: (i) pyrolyzing the acrylic emulsion to generate additional nanoporosity that suppresses the solid-phase heat conduction, (ii) eliminating combustible fractions to reduce the gross calorific value, and (iii) preserving the hydrophobic silica skeleton from the aerogel and potassium silicate components. This simultaneous pore formation, flammability reduction, and hydrophobicity retention distinguishes our approach from prior strategies. By systematically varying the acrylic-to-potassium silicate ratio, we investigated its influence on the microstructure, hydrophobicity, thermal stability, and insulation performance. The resulting composites feature hierarchical porosity (aerogel nanopores combined with hollow microsphere air pockets) and a fiber-reinforced skeleton, forming a synergistic thermal-barrier system that maintains low thermal conductivity while achieving markedly improved mechanical integrity. This facile and scalable approach offers a practical pathway for designing next-generation insulation materials tailored for aerospace, building envelopes, and industrial high-temperature applications requiring both thermal efficiency and long-term stability.

## 2. Results and Discussion

### 2.1. Microstructure

**Figure 1:**
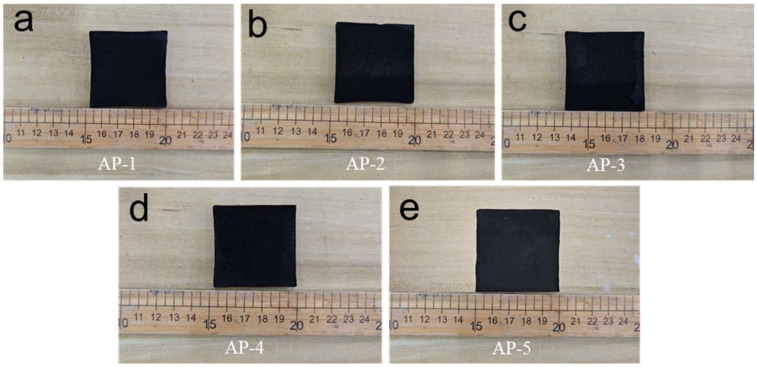
Photographs of samples: (**a**–**e**) digital photographs of AP-1 to AP-5. A ruler is provided for size reference.

**Figure 2:**
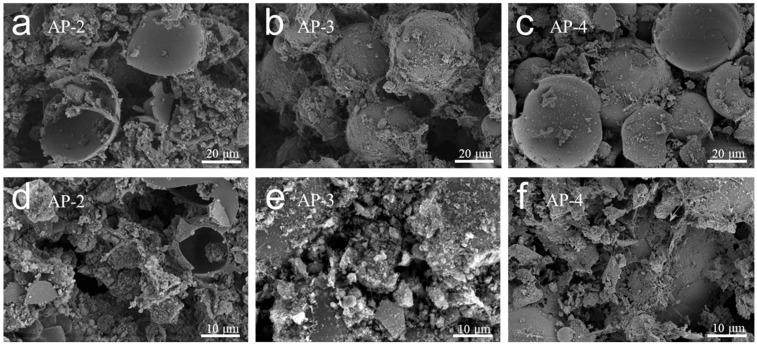
SEM morphologies of samples: (**a**–**c**) low-magnification and (**d**–**f**) high-magnification micrographs of AP-2, AP-3, and AP-4.

The size of the sample is about 5.0 × 5.0 × 0.5 cm, and it has a black surface, and there are no macroscopic holes or cracks (see Figure 1). The blackening likely results from carbonization of styrene-acrylic components during the high-temperature treatment. SEM images of AP-2, AP-3, and AP-4 (see Figure 2) reveal the porous network typical of aerogels and clearly show the hollow glass microspheres. At the 10 μm scale, the silica aerogel-derived particles are observed to be distributed on the surface; although some densification is evident due to ambient drying, the remnant nanoporous features are still discernible. At the 20 μm scale, the hollow glass microspheres appear uniformly embedded within the matrix, with many exhibiting a well-coated morphology, indicating good interfacial compatibility between the organic-inorganic hybrid matrix and the microspheres. Notably, despite the presence of the matrix coating, the hollow microspheres retain their integrity, and the aerogel-derived particles exhibit a partially retained porous architecture on the surface, collectively forming an intact multiscale thermal barrier structure.

On the scale of 10 µm, an unevenly distributed pore structure is evident. While the silica aerogel powder has undergone partial densification during ambient drying, the remnant porosity still contributes significantly to the composite’s overall pore volume. The aerogel greatly inhibits the heat transfer process by virtue of its ultra-high porosity, nano-scale pore size, and slender skeleton network [26,27]. On the scale of 20 µm, the obvious hollow or semi-hollow characteristics confirm the uniform dispersion of hollow glass microspheres and their role in providing bone support. The sealed hollow unit effectively prevents the heat transfer of gas, further reducing the contribution of gas to heat transfer [28].

**Figure 3:**
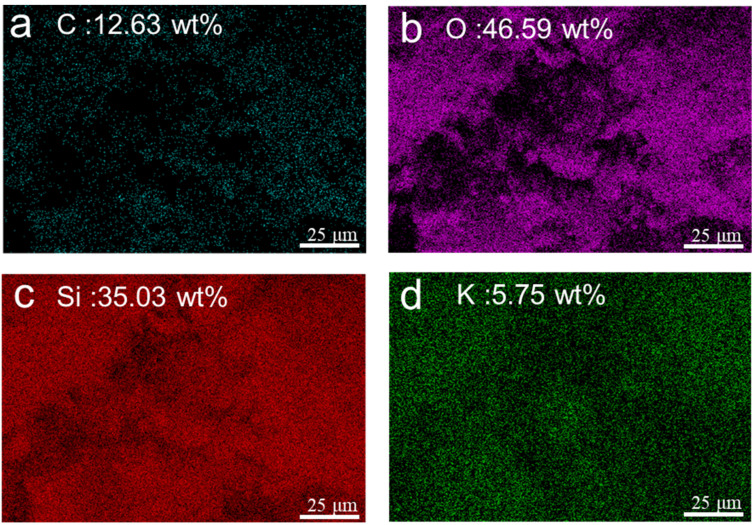
EDS elemental mapping of AP-3: (**a**–**d**) C, O, Si, K elemental distribution with corresponding weight percentages.

Elemental mapping and quantitative analysis of AP-3 are shown in Figure 3. The material contains 46.59 wt% O and 35.03 wt% Si, consistent with a preserved silica-oxygen network after carbonization. The O and Si originate primarily from the silica aerogel skeleton, the polycondensed potassium silicate matrix, and the incorporated glass fibers and hollow glass microspheres. The C content is only 12.63 wt%, which likely reflects decomposition and off-gassing of the organic constituents (mainly from the acrylic emulsion and silane coupling agent) during high-temperature carbonization, leaving a low residual carbon fraction. A relatively small amount of potassium is also detected, originating from the potassium silicate precursor.

### 2.2. Surface Chemistry and Composition

**Figure 4:**
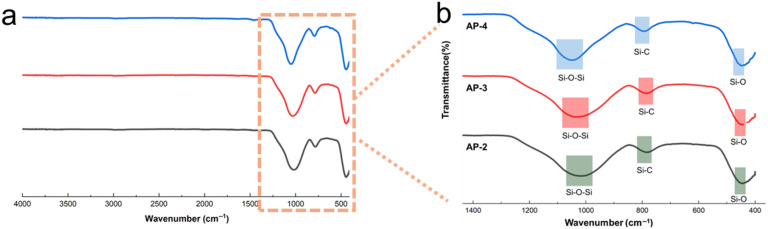
FTIR spectra of the samples: (**a**) full-wavenumber range (4000–500 cm^−1^) spectra of AP-2, AP-3, and AP-4; (**b**) magnified fingerprint region (1400–400 cm^−1^) with characteristic functional group assignments.

Figure 4 presents the FTIR spectra of the AP-2, AP-3, and AP-4 samples. All samples exhibit characteristic absorption bands at approximately 445 cm^−1^, 780–791 cm^−1^, and 1019–1049 cm^−1^, which are assigned to Si-O bending of the inorganic siloxane skeleton, Si-C stretching from methyl groups on the aerogel surface, and asymmetric Si-O-Si stretching, respectively [29,30,31]. The FTIR results indicate that the persistence of the Si-C band and main Si-O skeleton band shows that the surface methyl group and silica-oxygen network of aerogels are preserved after processing, which provides a chemical basis for the hydrophobicity of materials. Notably, the asymmetric Si-O-Si stretching band (~1019–1049 cm^−1^) exhibited only a marginal and gradual shift to lower wavenumbers as the acrylic emulsion fraction increased. The broad nature of this peak and the absence of any sharp features around 1080–1090 cm^−1,^ suggest that the siloxane network remains predominantly in a disordered, amorphous state, without detectable formation of strained 4-fold siloxane rings. Instead, the subtle red shift may reflect a slightly decreased average connectivity or a modified chemical environment of the inorganic network in the presence of the carbonaceous residues, rather than a structural re-ordering into distinct ring topologies. Furthermore, no distinct absorption bands are observed in the 3400–3500 cm^−1^ region (O-H stretching of hydrogen-bonded silanol groups) or at 950–960 cm^−1^ (Si-OH bending), indicating a low concentration of residual silanol groups in the composites. The near absence of these hydrophilic groups is consistent with the high water contact angles (>115° for all samples), confirming that the methyl-functionalized silica surface is well preserved and the condensation of the silanol groups during processing contributes to the hydrophobic character of the materials.

**Figure 5:**
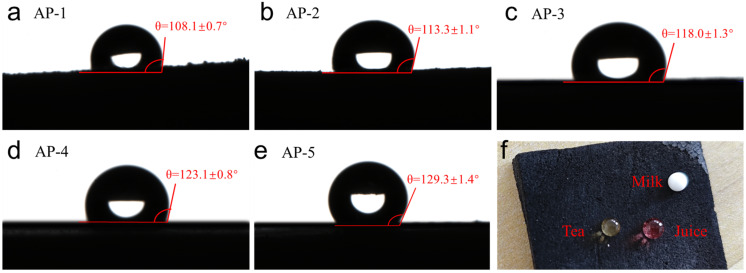
Hydrophobicity of samples: (**a**–**e**) water contact angle of AP-1 to AP-5; (**f**) liquid repellency behavior of the sample toward various liquids (milk, tea, and juice).

Figure 5 shows the water contact angles for the samples. All materials exhibit good hydrophobicity, and the contact angle increases with the decrease in the potassium silicate content. The pure acrylic emulsion sample (AP-5) reached the maximum contact angle of 129.3°, indicating good hydrophobicity. This trend stems from two synergistic effects: on the one hand, hydrophobic methyl groups on the surface of aerogels provide inherent hydrophobicity, and on the other hand, reducing potassium silicate reduces the surface density of hydrophilic groups, thus enhancing the wettability [32,33]. Notably, even the completely inorganic sample (AP-1) showed a contact angle of more than 115°, indicating that the hydrophobic groups anchored on the silica skeleton still endowed with remarkable water repellency [34,35]. Finally, Figure 5f shows the droplet morphology of milk, tea, and fruit juice on the sample surface, which further confirms the excellent antifouling performance of the composite material.

### 2.3. Thermal Analysis

**Figure 6:**
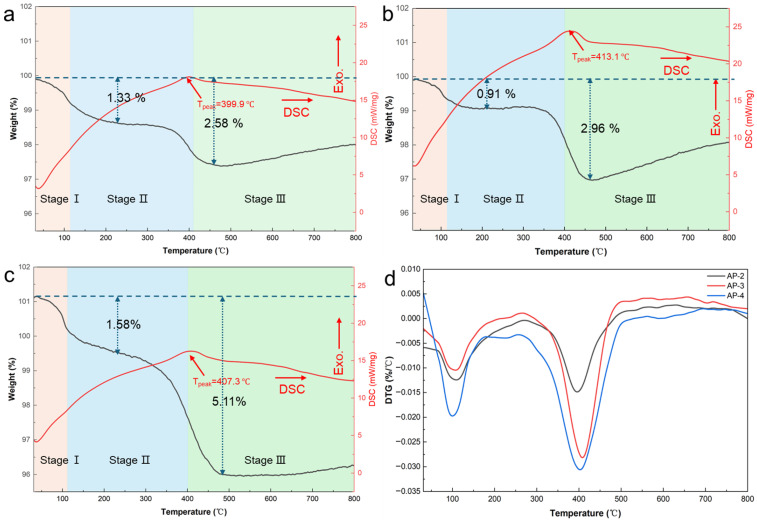
Thermal analysis of samples: (**a**–**c**) TG-DSC curves of AP-1, AP-2, and AP-3; (**d**) corresponding DTG curves.

Figure 6 shows the TG-DSC and DTG curves for AP-2, AP-3, and AP-4 measured in nitrogen from 30 °C to 800 °C at 10 °C/min. The thermal decomposition behavior of the samples is closely related to their ratio of acrylate emulsion to potassium silicate. All samples exhibit two main stages of weight loss. The first stage, occurring between 30 and 200 °C with a weight loss of approximately 1 wt%, is manifested as a gradual decline in the TG trace accompanied by a weak endothermic feature on the DSC curve. This behavior is attributed primarily to the desorption of the physically adsorbed water and volatilization of the residual coalescing agents. The second, more pronounced event is concentrated between 350 and 450 °C and shows a weight loss that increases with acrylate content (from 1 to 3 wt%). This high-temperature loss is consistent with thermal decomposition and oxidative release of organic constituents, such as residual acrylate fragments and alkyl groups from the silane coupling agents, as well as the further decomposition of silicone methyl groups on the surface of the silica aerogel. After heating to 800 °C, the residual mass consists mainly of thermally stable inorganic components, including the silica-oxygen skeleton derived from the potassium silicate and silica aerogel, as well as the potassium oxides formed during decomposition. These inorganic residues possess excellent high-temperature stability and oxidation resistance, providing the structural foundation for the composite’s thermal durability.

Figure 6d presents the DTG curves of the three samples. All three exhibit a two-stage weight loss profile, with AP-4 showing the highest weight loss rate in each stage. Specifically, the maximum weight loss rates for the first decomposition stage (30–200 °C) are 0.012%/°C for AP-2, 0.010%/°C for AP-3, and 0.020%/°C for AP-4. In the second main decomposition stage (350–450 °C), the peak values increase to 0.015%/°C, 0.028%/°C, and 0.031%/°C, respectively. In the low-temperature stage, the weight loss rates of AP-2 and AP-3 are relatively close; whereas in the high-temperature stage, the weight loss rate of AP-3 is higher than that of AP-2. Overall, with the increasing acrylate content, the maximum weight loss rate of the samples during the main decomposition stages shows an upward trend. This is in line with the earlier statement that the weight loss in these stages primarily stems from the thermal decomposition and oxidation of organic components.

Despite these weight losses, the total mass change up to 800 °C remains below 5 wt% for all samples, demonstrating excellent thermal stability. This resilience is attributable to the formation of a dense Si-O-Si inorganic network produced by high-temperature polycondensation of potassium silicate, which preserves the structural integrity and limits bulk degradation [36,37]. From an application perspective, the limited low-temperature desorption and modest high-temperature organic decomposition suggest that these composites are suitable for thermally demanding insulation or protection.

**Figure 7:**
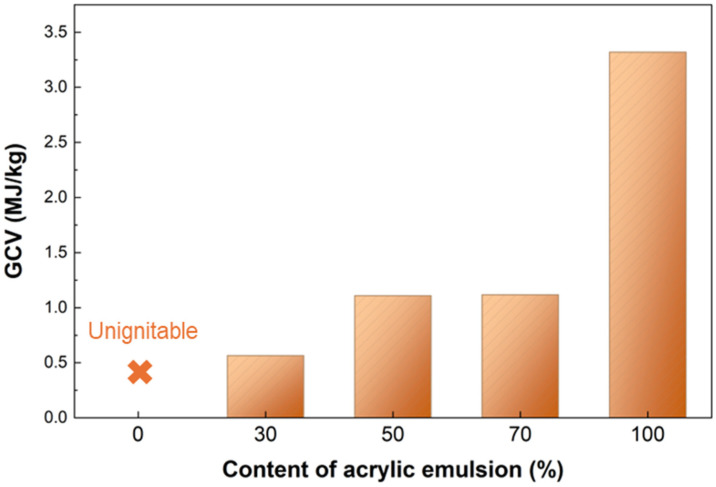
Combustion performance of samples: GCV (gross calorific value) of samples with different acrylic emulsion contents.

Figure 7 presents the GCV of the samples. The purely inorganic reference (AP-1) is essentially non-combustible, exhibiting a negligible GCV. As the acrylic-emulsion fraction increases, the GCV of the composites rises progressively. This increase is attributable to the larger organic (carbon-rich) fraction contributed by the acrylic emulsion: during thermal decomposition, the acrylic component produces combustible volatiles and carbonaceous residues that, upon oxidation, release heat and thereby raise the measured GCV. Furthermore, the inorganic component of potassium silicate can dilute the unit mass of the overall organic combustible components of the material, thereby further reducing the total combustion heat value of the sample. Notwithstanding this trend, the GCV of all samples remains low (<4.0 MJ/kg), substantially lower than typical engineering polymers or biomass fuels; this low calorific content indicates an intrinsically limited heat-release potential and, therefore, suggests favorable flame-resistance characteristics for the composite. Moreover, the high-temperature carbonization treatment employed during fabrication promotes the thermal decomposition of most organic components derived from the acrylic emulsion, thereby substantially reducing their original combustibility and yielding a final material with inherently low flammability. Taken together with the inorganic Si-O-Si network of the matrix, the low GCV supports the suitability of these materials for thermal-insulation applications where low heat release and flame resistance are required.

### 2.4. Heat Insulation Properties

**Figure 8:**
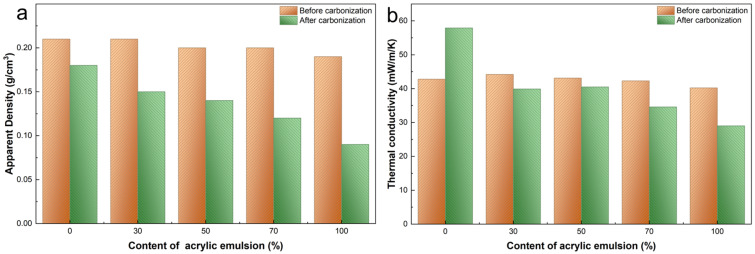
Physical and thermal properties of samples: (**a**) apparent density and (**b**) thermal conductivity of samples with different acrylic emulsion contents before and after carbonization.

Figure 8 illustrates the apparent density and thermal conductivity of the samples with varying acrylic-emulsion content, measured before and after carbonization. Both apparent density and thermal conductivity decrease markedly as the acrylic-emulsion fraction increases. Carbonization further reduces the apparent density of all samples. With the exception of the purely inorganic reference (AP-1), the thermal conductivity of the carbonized samples also declines, indicating that carbonization generally enhances the thermal insulation performance of the composites. The anomalous increase in thermal conductivity observed for AP-1 after carbonization is likely due to localized damage or densification of its inorganic framework during the high-temperature treatment.

All samples possess low apparent densities, which fall from 0.18 g/cm^3^ for AP-1 to 0.09 g/cm^3^ for AP-5. This trend is primarily ascribed to the larger organic fraction in higher-acrylic formulations: during high-temperature carbonization, a greater amount of organic material pyrolyzes and evacuates the matrix, causing weight loss and the development of a more open pore structure that lowers bulk density. Correspondingly, the thermal conductivity decreases continuously with increasing acrylic content, from 57.9 mW/(m·K) to 29.0 mW/(m·K), demonstrating a good insulating performance. The low thermal conductivity arises from a synergistic hierarchical porosity:remnant nanopores of the aerogel-derived phase suppress gaseous thermal conduction, while incorporated hollow glass microspheres increase macroporosity and obstruct conductive heat-flow paths [38,39]. In addition, carbonization of the acrylic phase produces a loose, porous carbonaceous structure that further reduces solid-phase thermal conduction. In summary, through high-temperature treatment and the regulation of the ratio of organic to inorganic components, which tailors the structural characteristics of the composites, the materials achieve both low thermal conductivity (29.0 mW/(m·K) at 0.09 g/cm^3^) thermal insulation and lightweight features while maintaining overall structural integrity.

**Figure 9:**
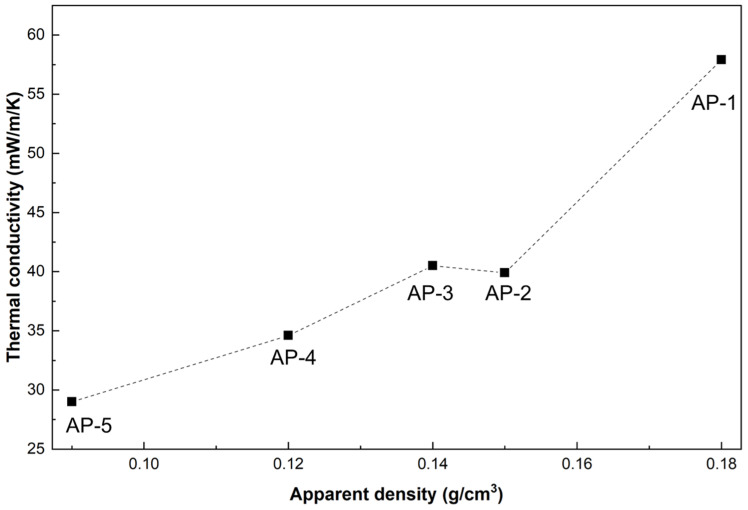
The thermal conductivity and apparent density of all samples.

Thermal conductivity is plotted against apparent density in Figure 9. An overall positive correlation is observed: as density decreases from 0.18 to 0.09 g/cm^3^, thermal conductivity drops from 57.9 to 29.0 mW/(m·K). A slight deviation occurs between AP-2 (0.15 g/cm^3^, 39.9 mW/(m·K)) and AP-3 (0.14 g/cm^3^, 40.5 mW/(m·K)), where thermal conductivity marginally increases despite the density reduction. This likely reflects the competing effects at intermediate acrylic contents, where carbonaceous residues with higher intrinsic conductivity partially offset the gain from increased porosity. At higher acrylic fractions, nanoporosity development dominates, restoring the decreasing trend. Combined with SEM observations (Figure 2), the disrupted solid networks confirm that the multiscale porous architecture effectively suppresses heat conduction by eliminating continuous thermal pathways.

Table 1 compares the apparent density, thermal conductivity, and water contact angle of the AP series composites with representative hydrophobic aerogel systems from recent literature. At a density of 0.09 g/cm^3^, AP-5 achieves a thermal conductivity of 29.0 mW/(m·K), which is among the lowest reported for hydrophobic aerogel composites in this density range. For comparison, a PI/TPU-0.5 aerogel with a similar density (0.10 g/cm^3^) exhibits a slightly higher thermal conductivity of 29.51 mW/(m·K) but a lower contact angle (123.6°). The SEP/SA composite at a density of 0.22 g/cm^3^ shows a lower thermal conductivity (26.1 mW/(m·K)), yet its density is more than twice that of AP-5, indicating that its enhanced insulation performance is achieved at the expense of lightweighting. These comparisons demonstrate that the AP composites, particularly AP-5, achieve a favorable balance of ultralow density, low thermal conductivity, and robust hydrophobicity. This balanced performance is attributed to the multiscale porous architecture formed by the synergistic integration of hollow glass microspheres, silica aerogel powders, and the carbonized hybrid matrix, which disrupts both solid and gaseous heat conduction pathways without requiring excessive density reduction.

The beneficial role of carbonization in achieving this performance is supported by multiple lines of experimental evidence. The TG-DSC analysis of the carbonized composites (Figure 6) shows a total weight loss below 5 wt% up to 800 °C, demonstrating that the carbonization step has effectively pre-eliminated thermally labile organic fractions or converted them into a thermally stable carbonaceous form, thereby ensuring high-temperature dimensional stability of the final product. The GCV data (Figure 7) confirm that the carbonized composites exhibit low gross calorific values below 4.0 MJ/kg, indicating intrinsically limited heat-release potential and reduced fire hazard. SEM observations (Figure 2) reveal that the carbonized acrylic phase generates additional porous structures throughout the matrix, which, together with the residual porosity from the silica aerogel powder, create a hierarchical pore network that elongates solid-phase heat conduction paths. The combined structural and compositional effects are reflected in the simultaneous decrease in apparent density and thermal conductivity with increasing acrylic content (Figure 8), where more acrylic emulsion leads to more extensive carbonization-induced porosity and correspondingly lower thermal conductivity. Taken together, these complementary results establish that carbonization functions as a critical structure-directing step that concurrently generates supplementary porosity, reduces combustible content, and contributes to the overall thermal insulation performance.

**Figure 10:**
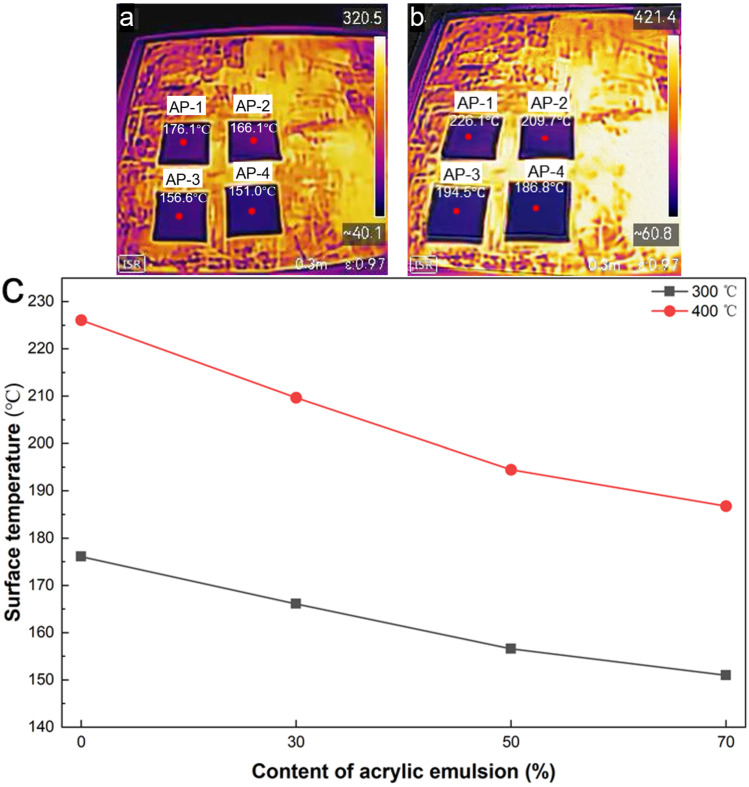
Thermal insulation performance of samples: infrared thermograms of the backside surface of 5 mm thick samples after unilateral heating for 10 min on a heating plate at 300 °C (**a**) and 400 °C (**b**), and surface temperature comparison (**c**).

Figure 10 presents the infrared thermogram of a 5 mm thick sample after unilateral heating for 10 min on heating plates at 300 °C and 400 °C. Under two conditions, the measured surface temperature decreased systematically with the increase in acrylic emulsion fraction. At 300 °C, the surface temperatures of AP-1, AP-2, AP-3, and AP-4 are 176.1 °C, 166.1 °C, 156.6 °C and 151.0 °C, respectively, indicating that the formula with higher acrylic emulsion content has better thermal insulation performance, which is consistent with the thermal conductivity measurement results. The obvious temperature difference between the heated surface and the exposed surface further proves that the porous structure of the composite materials effectively hinders heat transfer. At 400 °C, the absolute surface temperature rises moderately, but with the increase in acrylic emulsion content, the same downward trend persists, indicating that the composites maintain good thermal insulation stability at high temperature.

### 2.5. Mechanical Properties

**Figure 11:**
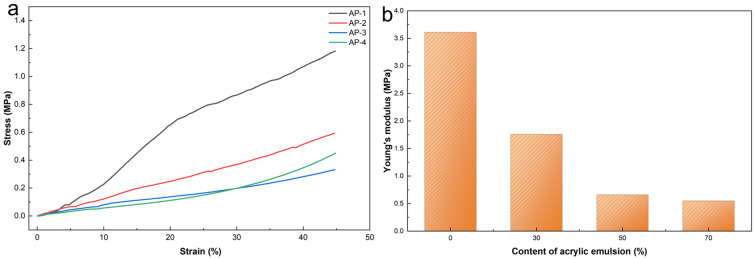
Mechanical properties of samples: (**a**) stress–strain curves of AP-1 to AP-4; (**b**) Young’s modulus of samples with different acrylic emulsion contents.

To evaluate the influence of the acrylic emulsion content on the structural integrity of the composites, uniaxial compression tests were conducted. Figure 11a presents the compressive stress–strain curves of the samples with varying acrylic emulsion content. All specimens exhibit a monotonically increasing stress throughout the entire strain range, without any descending branch or distinct yield plateau, which is characteristic of the progressive densification in porous composite materials. The purely inorganic sample (AP-1) shows the steepest initial slope and maintains the highest stress at any given strain level. As the acrylic content increases, the stress–strain curves become progressively shallower, reflecting a reduction in structural rigidity and enhanced deformability. This transition is attributed to the increasing proportion of carbonaceous residues derived from the acrylic emulsion after carbonization, which form a more compliant porous network compared to the rigid Si-O-Si skeleton from potassium silicate. Despite the reduction in stiffness, all samples maintain structural integrity during compression without catastrophic failure, suggesting that the hybrid porous architecture and the reinforcing effect of glass fibers and hollow microspheres effectively suppress the brittle fracture.

Correspondingly, the Young’s modulus derived from the initial linear portion of each stress–strain curve progressively decreased with increasing acrylic fraction. As shown in Figure 11b, the Young’s modulus progressively decreased with increasing acrylic fraction. The purely inorganic sample (AP-1, 0% acrylic) exhibited the highest modulus of 3.61 MPa. This value dropped sharply to 1.76 MPa at 30% acrylic content (AP-2) and further declined to 0.66 MPa at 50% (AP-3). At 70% acrylic content (AP-4), the modulus decreased slightly to 0.55 MPa.

This decreasing trend can be attributed to the trade-off between the rigid inorganic Si-O-Si network and the softer carbonaceous residue derived from the carbonization of the acrylic emulsion. At low acrylic content, the potassium-silicate-derived inorganic framework dominates, imparting higher stiffness. This is attributed to the dehydration condensation of silanol groups (Si-OH) in potassium silicate at elevated temperatures, which results in the formation of a dense and highly rigid silicon ceramic layer [43,44]. As the organic fraction increases, a more porous carbon structure forms during pyrolysis, reducing the overall mechanical rigidity of the composite. Notably, even at 70% acrylic content, the modulus remained above 0.5 MPa, indicating that the hollow microspheres continue to contribute to structural integrity. These results suggest that while increasing acrylic content enhances hydrophobicity and thermal insulation (as discussed in Section 2.2 and Section 2.4), it compromises mechanical stiffness. Therefore, an optimal balance must be achieved depending on the target application. Although the modulus decreases from 3.61 MPa to 0.55 MPa, it should be noted that the density simultaneously decreases by 50% (from 0.18 to 0.09 g/cm^3^). When normalized by density, the specific modulus of AP-4 (~4.6 MPa·cm^3^/g) indicates that the intrinsic structural efficiency is partially preserved. For thermal insulation applications where the primary mechanical requirement is handling and installation integrity rather than load-bearing, a modulus above 0.5 MPa is generally considered sufficient. For comparison, conventional fiberglass insulation batts exhibit compressive moduli in the range of 0.1–1.0 MPa. Thus, while the stiffness reduction represents a genuine trade-off with lightweighting, the mechanical performance remains adequate for the intended insulation applications.

## 3. Conclusions

In this study, an organic-inorganic synergistic composite route was proposed, and silica aerogel-containing composites reinforced by the chopped glass fibers and uniformly dispersed hollow glass microspheres were prepared by the high-temperature carbonization process. The effects of the ratio of the acrylic emulsion to potassium silicate on the microstructure, hydrophobicity, thermal stability, and thermal insulation properties of the materials were systematically investigated. The experimental results show that with the increase in the content of acrylic emulsion, the surface hydrophobicity of the material is significantly improved (the maximum water contact angle is 129.3°). At the same time, the apparent density of the sample can be reduced from 0.18 g/cm^3^ to 0.09 g/cm^3^, and the thermal conductivity can be reduced from 57.9 mW/(m·K) to 29.0 mW/(m·K), which shows effective ultra-light heat insulation characteristics. Micro-characterization shows that the hierarchical multi-scale pore structure (the cooperative construction of the nano-scale aerogel network and macro-hollow microspheres) and the porous carbonaceous phase formed after carbonization jointly inhibit the heat transfer between the gas phase and solid phase, thus realizing the simultaneous decrease in thermal conductivity and density and enhancing the surface hydrophobicity. Notably, the carbonization step plays a central role in simultaneously achieving three critical functions: generating supplementary porosity from the acrylic-derived phase, reducing combustible content, and preserving the hydrophobic silica framework. This triple functionality distinguishes the present strategy from conventional single-purpose carbonization treatments. Thermogravimetric analysis further proved that the total weight loss of each sample was less than 5% at 800 °C, indicating excellent high-temperature thermal stability. Furthermore, mechanical testing revealed that the Young’s modulus decreased with increasing acrylic content, from 3.6 MPa for the purely inorganic sample to 0.55 MPa at 70% acrylic content, reflecting the trade-off between stiffness and organic-derived porosity. Generally speaking, the silica aerogel-containing composite system with organic-inorganic cooperation and high-temperature carbonization constructed in this work shows comprehensive advantages in taking into account the ultra-low thermal conductivity, remarkable hydrophobicity, good mechanical integrity, and high-temperature stability and provides a feasible material design and preparation path for the next generation of high-temperature energy efficiency systems, building insulation, and special thermal protection fields such as industry and aerospace.

## 4. Materials and Methods

### 4.1. Raw Materials

The acrylic emulsion (solid content: 47 ± 1%) and silane coupling agent KH550 (purity: >99%) were purchased from Shandong Yousuo Chemical Co., Ltd. (Linyi, China). Potassium silicate solution with a solid content of 30 wt% and a modulus (SiO_2_/K_2_O molar ratio) of 3.2 was purchased from Fu Fei Chemical Industry Co., Ltd. (Guangzhou, China). Industrial-grade silica aerogel powders were purchased from Sinochem Hualu New Material Co., Ltd. (Chongqing, China). Glass fibers were purchased from Hongyao Mineral Products Processing Co., Ltd. (Shijiazhuang, China). Hollow glass microspheres were purchased from Zhongke Huaxing New Materials Co., Ltd. (Taizhou, China). The film-forming agent (purity: >99%) was purchased from Juying Chemical New Materials Co., Ltd. (Shanghai, China). Deionized water (DI⋅H_2_O) was produced in-house using a high-purity water system (model ECO-S, HHitech, Shanghai, China).

### 4.2. Samples Preparation

**Figure 12:**
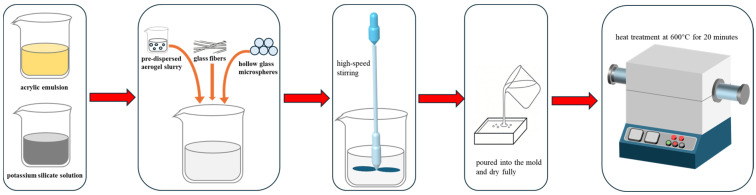
Preparation process of samples: schematic illustration of the fabrication of AP series composites.

First, the acrylic emulsion was mixed with the potassium silicate solution at weight ratios (*w*/*w*) of 0:10, 3:7, 5:5, 7:3 and 10:0 were designated as AP-1, AP-2, AP-3, AP-4, AP-5. Subsequently, the pre-dispersed aerogel slurry, glass fibers, and hollow glass microspheres were incorporated sequentially. During this process, a silane coupling agent and a film-forming additive were added. The mixture was then homogenized via high-speed stirring for 10 min, poured into a mold, and allowed to dry fully for 24 h at 35 °C. In addition to the acrylic emulsion and potassium silicate at the specified ratios, each formulation contained 16 wt% silica aerogel powder, 20 wt% hollow glass microspheres, 2 wt% chopped glass fibers, 2 wt% silane coupling agent (KH550), and 4 wt% film-forming agent. After demolding, the resulting monolith was transferred to a tube furnace. The furnace was sealed and purged with flowing argon (purity ≥ 99.99%, flow rate 100 mL/min) for 15 min to remove residual air. The sample was then heated from room temperature to 600 °C at a ramp rate of 5 °C/min under continuous argon flow, held at 600 °C for 20 min, and naturally cooled to room temperature under argon before removal (Figure 12).

### 4.3. Testing and Characterization Methods

#### 4.3.1. Physical Properties

The obtained samples were ground to a smooth finish using sandpaper. Subsequently, the length, width, and heights at the midpoints of the four edges were measured. The volume was derived from these measurements, and the apparent density of the samples was calculated accordingly.

The water contact angle (WCA) was measured using a contact angle goniometer (model ASR-705S, Guangdong Aisry Instrument Technology Co., Ltd., Dongguan, China) with the average value calculated from three measurements per sample.

#### 4.3.2. Thermophysical Properties Testing

The thermal conductivity of different samples was measured by the transient hot wire method using a thermal conductivity meter (TC3000E, XIATECH, Xi’an, China), with the average value calculated from three measurements. The measurement procedure followed ASTM C1113/C1113M.

The temperature distribution under a steady-state heat flux was measured using an infrared thermal imaging camera (E09PRO, Hangzhou, China).

The thermal stability analysis was performed using TG-DSC (STA8000-FTIR-GCMS-ATD, Shanghai, China) with a heating rate of 10 °C/min from room temperature to 800 °C under nitrogen.

The gross calorific value (GCV) of the samples was determined using a bomb calorimeter (AM-C1009, Changsha Yuanfa Instrument Co., Ltd., Changsha, China).

#### 4.3.3. Microstructural Characterization

The microscopic pore structure of the sample surface was observed using scanning electron microscopy (Sigma 300, Zeiss, Oberkochen, Germany). Prior to imaging, small specimens (~5 mm × 5 mm) were cut from the bulk monoliths, mounted on aluminum stubs with conductive carbon tape, and sputter-coated with a thin layer of gold to minimize charging effects.

#### 4.3.4. Chemical Structural Analysis

Functional group changes were analyzed by Fourier-transform infrared spectroscopy (FTIR, Thermo Nicolet iS50, Thermo Fisher Scientific, Waltham, MA, USA) in the attenuated total reflectance mode over the range of 4000–400 cm^−1^ with 32 scans at a 3.818 cm^−1^ resolution.

#### 4.3.5. Mechanical Property Testing

The compressive Young’s modulus of the samples was determined using a universal testing machine (WHY-200, Shanghai Hualong Test Instruments Co., Ltd., Shanghai, China) at a constant loading rate of 0.5 mm/min. The modulus was determined from the linear elastic region of the stress–strain curve.

## Figures and Tables

**Table 1:** Comparison of apparent density, thermal conductivity, and water contact angle with previously reported hydrophobic aerogel composites.

Sample	Apparent Density (g/cm^3^)	Thermal Conductivity (mW/m·K)	Water Contact Angle (°)	Ref.
315C-PNAC	0.28	54.3	141	[40]
46.0% SEP/SA	0.22	26.1	121	[29]
GFWA-30	0.0973	35.3	125.8	[41]
PI/TPU-0.5 aerogel	0.10	29.51	123.6	[42]
AP-5	0.09	29.0	129.3	This work

## Data Availability

The original contributions presented in this study are included in the article. Further inquiries can be directed to the corresponding author.
